# Solitary pulmonary nodule and ^18^F-FDG PET/CT. Part 1: epidemiology, morphological evaluation and cancer probability*

**DOI:** 10.1590/0100-3984.2014.0012

**Published:** 2016

**Authors:** Marcos Pretto Mosmann, Marcelle Alves Borba, Francisco Pires Negromonte de Macedo, Adriano de Araujo Lima Liguori, Arthur Villarim Neto, Kenio Costa de Lima

**Affiliations:** 1 Master, Nuclear Physician at Liga Norte Riograndense Contra o Câncer, Natal, RN, Brazil.; 2 MDs, Radiologists at Liga Norte Riograndense Contra o Câncer, Natal, RN, Brazil.; 3 PhD, Nuclear Physician at Liga Norte Riograndense Contra o Câncer, Natal, RN, Brazil.; 4 Post Doc Fellow, Professor, Programa de Pós-Graduação em Saúde Coletiva -Universidade Federal do Rio Grande do Norte (UFRN), Natal, RN, Brazil.

**Keywords:** Solitary pulmonary nodule, Positron-emission tomography, Computed tomography

## Abstract

Solitary pulmonary nodule corresponds to a common radiographic finding, which is frequently detected incidentally. The investigation of this entity remains complex, since characteristics of benign and malignant processes overlap in the differential diagnosis. Currently, many strategies are available to evaluate solitary pulmonary nodules with the main objective of characterizing benign lesions as best as possible, while avoiding to expose patients to the risks inherent to invasive methods, besides correctly detecting cases of lung cancer so as the potential curative treatment is not delayed. This first part of the study focuses on the epidemiology, the morfological evaluation and the methods to determine the likelihood of cancer in cases of indeterminate solitary pulmonary nodule.

## Abstract (pt)

O nódulo pulmonar solitário corresponde a um achado radiológico comum, cuja detecção ocorre frequentemente de forma incidental. A investigação desta entidade permanece complexa, uma vez que existem sobreposições entre as características dos processos benignos e malignos no seu diagnóstico diferencial. Atualmente, muitas estratégias estão disponíveis para a avaliação do nódulo pulmonar solitário, sendo que o objetivo principal consiste em caracterizar da melhor forma possível as alterações benignas, não expondo os pacientes aos riscos de métodos invasivos, e detectar corretamente os casos de câncer de pulmão, não retardando potencial tratamento curativo. Esta primeira parte do estudo tem como foco apresentar a epidemiologia, revisar a avaliação morfológica e demonstrar métodos para estimar a probabilidade de câncer em nódulo pulmonar solitário indeterminado.

## INTRODUCTION

Solitary pulmonary nodule is a single radiological, round, well circumscribed opacity with ≤ 3 cm in diameter. It is characterized by being completely surrounded by pulmonary parenchyma, and is not associated with atelectasis, lymph node enlargement, pneumonia and pleural effusion^(1)^. Lesions are subdivided according their size, so lesions < 8-10 mm (subcentimeter nodules) present with lower probability of malignancy and have different recommendations for investigation as compared with larger solitary pulmonary nodules^(2)^.

The classical definition of indeterminate solitary pulmonary nodule - a potentially malignant lesion - refers to pulmonary nodules that do not meet the typical radiological criteria of benignity^(3)^.

The term "pulmonary mass" is currently utilized for pulmonary lesions > 3 cm in diameter, whose likelihood of malignant disease is considerably increased^(2)^.

## PREVALENCE AND INCIDENCE

Most solitary pulmonary nodules are incidentally detected at chest radiography and computed tomography (CT) requested to investigate other diseases. Approximately 150,000 solitary pulmonary nodules are detected every year in the United States of America^(4)^. It is estimated that the frequency of solitary pulmonary nodules in Brazil is high, considering the high rates of lung cancer and of infectious diseases. A population study developed in 1959^(5)^ demonstrated the presence of one solitary pulmonary nodule per every 500 chest radiographs (0.2%).

A study developed by The Early Lung Cancer Action Project included 1,000 volunteers of a North American population at high risk for lung cancer, submitted to chest radiography and CT. Noncalcified nodules were detected at lowdose CT in 23% of the individuals. Chest radiography presented positive results in 7% of cases, and approximately half of such cases corresponded to false-positive results. Malignant disease was diagnosed in 2.7% of cases^(6)^.

The prevalence of cancer varies a lot, according to the evaluated population or subgroup^(7)^. In studies utilizing 18-fluorodesoxyglucose (^18^F-FDG) positron emission tomography (PET), the malignancy prevalence may range between 46% to 82%^(7)^. In screening studies, the prevalence of malignancy is much lower, ranging between 2% and 13% of cases^(7)^.

## DIFFERENTIAL DIAGNOSIS

The first step in the evaluation is to determine whether the abnormality actually corresponds to a solitary pulmonary nodule.

At chest radiography, about 20% of the "suspicious nodules" may in truth be associated with alterations mimicking solitary pulmonary nodules^(4)^. Amongst the main causes one can mention the following: ribs fractures; sclerotic bone lesions; skin lesions (hemangiomas, warts, lipomas, neurofibromas), electrodes and nipples^(4)^.

There is a range of entities which manifest as solitary pulmonary nodules at chest radiography and CT, extending the possibilities of differential diagnoses, including mainly neoplastic lesions (both benign and malignant); inflammatory lesions (infectious and noninfectious); vascular and congenital lesions (Table 1)^(4)^.

**Table 1:** Differential diagnosis of solitary pulmonary nodule

Malignant neoplasms	Bronchogenic carcinoma
	Carcinoid tumor
	Pulmonary lymphoma
	Pulmonary sarcoma
	Solitary metastases
Benign neoplasms	Hamartoma
	Adenoma
	Lipoma
Infectious inflammatory	Granuloma (tuberculous/fungal)
	Nocardia infection
	Round pneumonia
	Abscess
Non infectious inflammatory	Rheumatoid arthritis
	Wegener's granulomatosis
	Sarcoidosis
Vascular	Arteriovenous malformation
	Infarction
	Hematoma
Congenital	Bronchial atresia
Miscelaneous	External object
	Pseudotumor
	Pleural thickening

Adapted from Erasmus et al.^(4)^

The main causes of malignant diseases include: adenocarcinomas (47%); squamous cell carcinoma (22%); solitary metastases (8%); undifferentiated non-small cell carcinoma (7%), small cell lung cancer (4%); and adenocarcinoma in situ (formerly called bronchioalveolar carcinoma) (4%). Amongst the less frequent causes, one can mention large cell carcinomas; carcinoid tumors; intrapulmonary lymphomas; adenosquamous carcinomas and malignant teratomas^(7)^.

The main causes of benign disease correspond either to residual or nonspecific granulomas (25%), infectious granulomas (15%), and hamartomas (15%). Less frequent causes of benign nodules include inflammation, fibrosis, pulmonary abscesses, round pneumonia, athelectasis, bronchogenic cysts, residual pulmonary infarction, focal hemorrhage, hemangioma and arteriovenous malformations^(7)^.

Such data correspond to studies evaluating solitary pulmonary nodules with ^18^F-FDG PET, most of them with North American populations^(7)^.

## MORPHOLOGICAL CHARACTERISTICS

The evaluation of morphological characteristics specific of solitary pulmonary nodules at conventional imaging studies is essential for appropriate investigation of patients^(8)^.

### Size

The size of a solitary pulmonary nodule is a relevant factor to assist in the differentiation between benign and malignant processes. As a general rule, larger nodules present higher probability of cancer^(8)^.

The probability of cancer varies a lot with the size of nodules in the different studied populations. Approximately 80% of benign nodules are < 2 cm in diameter. However, 15% of the malignant nodules are < 1 cm, and approximately 42%, < 2 cm^(4)^.

### Growth

An essential parameter in cases of solitary pulmonary nodule is the determination of the lesion growth rate, which can be obtained by comparing serial chest radiographs or CT. As a nodule doubles in volume it corresponds to a 26% increase in diameter^(3)^. As a nodule doubles in diameter, there is a eight-fold increase in volume^(9)^.

The time spam a malignant nodule takes to double in size is highly variable, generally ranging between 20 and 300 days^(7)^. Stability over a two-year period implies a doubling time of at least 730, strongly suggesting benignity^(3)^. Although two-year stability is widely accepted, some authors have questioned its validity as a predictive factor for benignity^(10)^, therefore, a longer follow-up should be considered in the subgroup of patients who present ground glass opacity at CT, since they may be associated with slow-growing adenocarcinoma in situ (bronchoalveolar cancer)^(7)^.

It is formally recommended that, provided there is no contraindication, a histological sample be obtained from solitary pulmonary nodules with evidenced growth at imaging studies^(7)^.

### Margins

**Figure 1:**
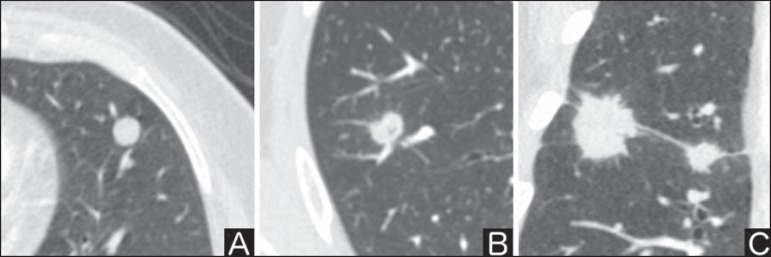
*Margins.* Chest CT - lung window. **A:** Regular contours and and smooth margins. **B:** Lobulated contours. **C:** Irregular contours.

Nodules margins and contours are classified into smooth, lobulated or irregular (Figure 1). There is a strong association between such a variable and cancer probability^(4)^.

Smooth margin is not indicative of benignity, considering that up to one third of malignant lesions present with such characteristic. Lobulated margin corresponds to a nodule with different growth rates, and in approximately 40% of cases is associated with a malignant process. Irregular margin has a strong predictive value for malignancy (approximately 90%)^(8)^.

Although irregular margins are strongly suggestive of a malignant process, they may occasionally be secondary to other alterations such as, for example, granulomatous disease, organizing pneumonia and progressive massive fibrosis^(8)^.

### Location

The location of a solitary pulmonary nodule in the pulmonary parenchyma is quite variable, since both benign and malignant conditions may manifest in any of the lung lobes^(8)^.

However, some location patterns may be observed in cases of lung cancer. Studies have demonstrated that approximately 70% of malignant lung tumors are located in the upper lobes and, also, primarily in the right lung^(11,12)^. Additionally, 50% of primary adenocarcinomas generally manifest as a peripheral solitary pulmonary nodule, while squamous cell carcinoma most frequently manifests as a centralized lesion^(13)^.

### Calcification

Calcification is the main radiological characteristic for differentiation between malignant and benign solitary pulmonary nodules^(4)^.

**Figure 2:**
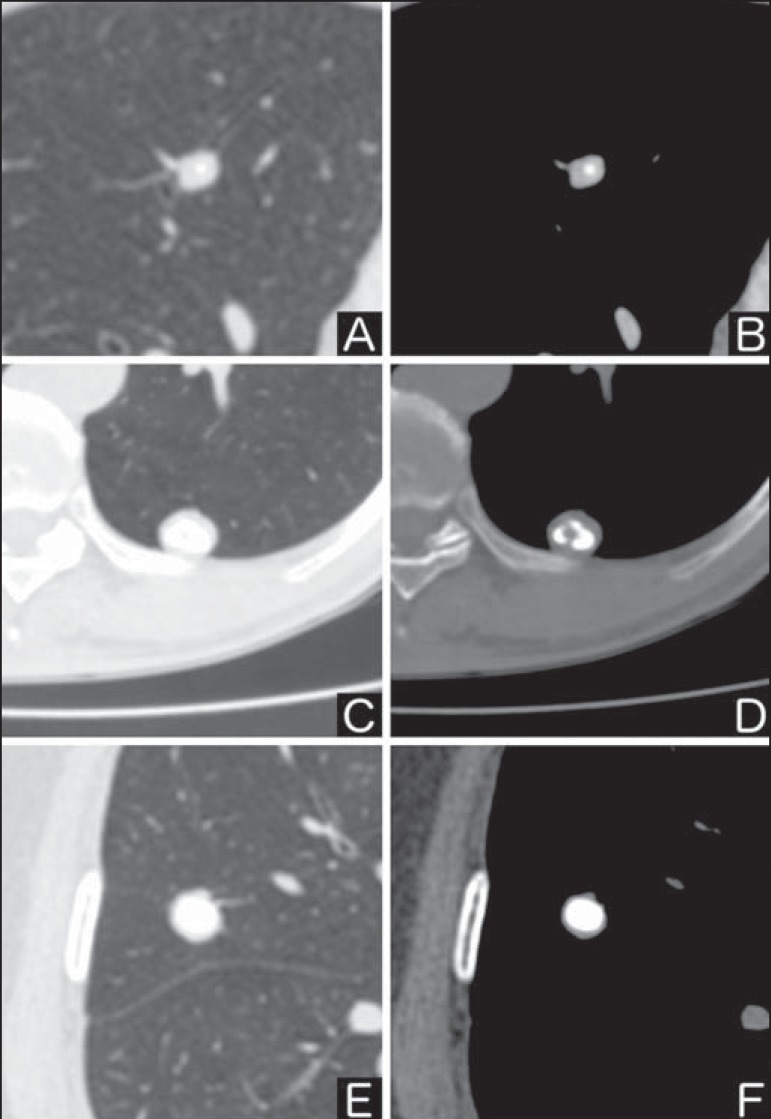
*Benign calcification pattern.* Chest CT - lung and bone windows. **A,B:** Central calcification. **C,D:** Popcorn calcification. **E,F:** Diffuse calcification.

The benign calcification pattern corresponds to central distribution, laminated, popcorn-like or diffuse (Figure 2). Solitary pulmonary nodules with such characteristics present with about 100% benignity probability^(8)^. Popcorn calcification is observed in up to one third of hamartomas, while other patterns are frequently found in cases of granulomatous infections, such histoplasmosis or tuberculosis^(4)^.

**Figure 3:**
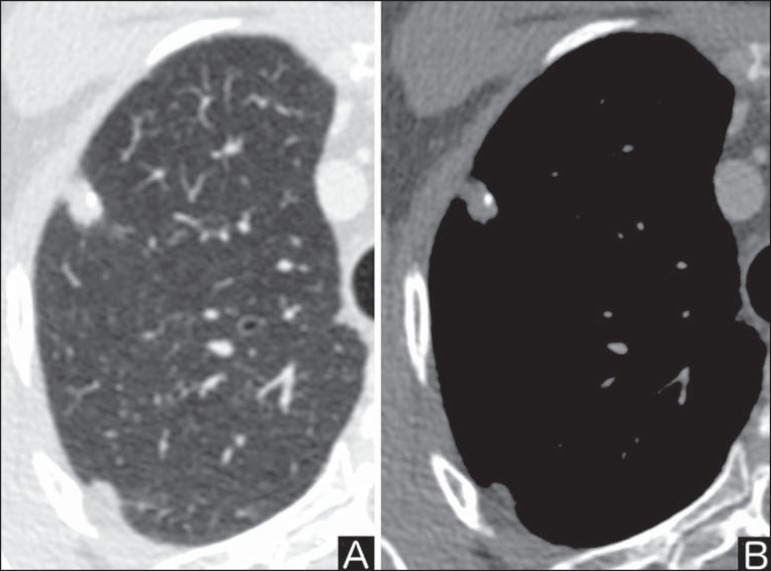
*Malignant calcification pattern.* Chest CT - lung window (**A**) and mediastinal window (**B**). Eccentric calcification.

Some studies have demonstrated that up to 13% of malignant lung tumors may present with some degree of calcification, but such rate decreases to only 2% for lesions < 3 cm in diameter^(8)^. The radiological patterns of eccentric and stippled calcifications increase the malignancy likelihood^(1)^ (Figure 3). Such characteristics might represent a malignant lesion involving a benign calcified nodule or even a malignant process with distrophic calcification^(4)^. Special situations to be considered include patients with metastatic carcinoid tumors or osteosarcoma and chondrosarcoma whose calcification pattern may be variable^(8)^.

Plain chest radiography has sensitivity, specificity and positive predictive value to identify calcification of respectively 50%, 87% and 93%, as compared with chest CT^(1)^.

### Fat

**Figure 4:**
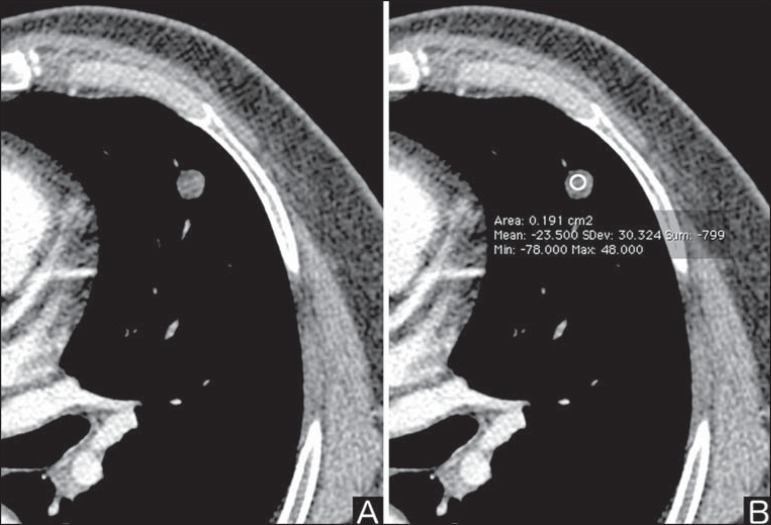
*Fat.* Presence of intranodular fat in hamartoma. Chest CT - mediastinal window (**A,B**). On **B**, observe the region of interest demonstrating median attenuation of -23 HU.

The presence of intranodular fat increases the probability of benignity, with hamartoma and lipoma (less frequently) being the main causes to be taken into consideration (Figure 4). Eventually, some malignant processes may present such characteristic, particularly metastases from liposarcoma or renal cell carcinoma^(4,8)^. About 50% of hamartomas assessed by chest CT present with fat inside^(14)^.

### Attenuation

**Figure 5:**
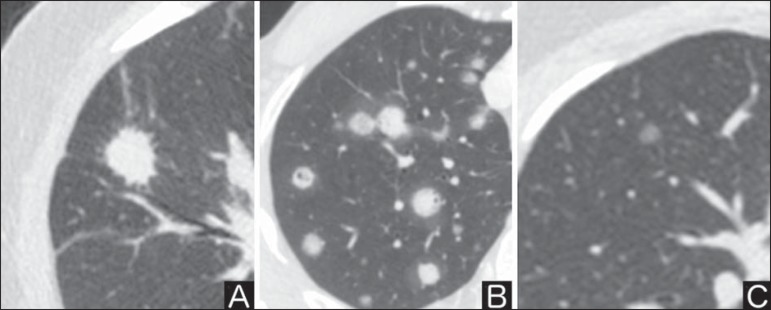
*Attenuation.* Chest CT - lung window. **A:** Solid. **B:** Partially solid. **C:** Non solid.

On the basis of chest CT findings, solitary pulmonary nodules can be classified into solid, partially solid and nonsolid^(15)^ (Figure 5).

A population-based screening study involving North-American individuals with high risk for lung cancer evaluated the frequencies of type of nodules attenuation, correlating them with the final diagnosis of malignancy. Amongst 233 positive findings, 81% were solid nodules, 7% were partially solid, and 12% were non solid, and the malignancy frequency corresponded to 32%, 63% and 13% of the respective nodules^(16)^.

### Air bronchogram

**Figure 6:**
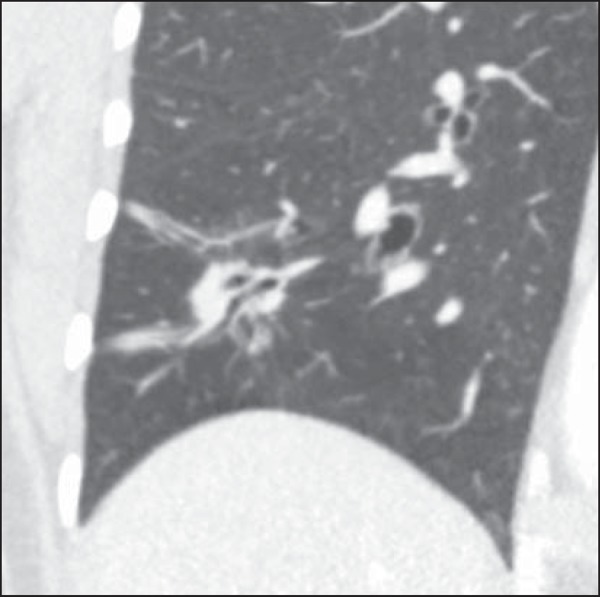
*Air bronchogram*. Chest CT - lung window. Nodule with lobulated contours intermingled with air bronchograms in the right lower lobe.

The radiological finding defined as air bronchogram is more frequently observed in cases of malignant lung tumors than in cases of benign nodules^(14)^ (Figure 6). Such characteristic, also called tubular transparency or pseudocavity, is found in up to 55% of adenocarcinomas in situ (bronchioalveolar carcinomas)^(8)^. However, other conditions may also present with such finding, for example, lymphoma, organizing pneumonia, pulmonary infarction and sarcoidosis^(9)^.

### Excavation (cavity)

**Figure 7:**
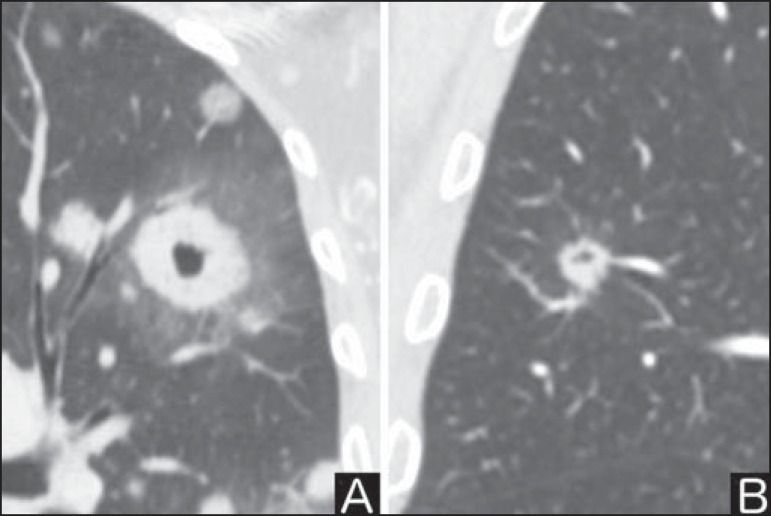
*Excavation.* Chest CT - lung window. **A:** Multiple pulmonary nodules, the largest one, with central excavation. **B:** Nodule with irregular contours, with small excavation.

Excavation may be found in benign and malignant solitary pulmonary nodules (Figure 7). Frequently, this is a finding associated with major lesions, but it can be visualized in small nodules of up to approximately 7 mm in diameter^(8)^. A study^(17)^ has demonstrated that the excavation wall thickness may be useful in the differential diagnosis, since only 5% of all nodules with thin walled cavity (< 5 mm) were malignant, while the malignancy likelihood increased to 85% in nodules with greater wall thickening (> 15 mm).

## CANCER PROBABILITY

The great diagnostic challenge in the evaluation of a patient with solitary pulmonary nodule is to securely establish if the nodule is benign or malignant. In a young patient with a solid, calcified, well defined solitary pulmonary nodule measuring 0.4 cm and stable for more than two years, the probability of a benign process is extremely high. On the other hand, an elderly, smoking, patient with a non-calcified, spiculated pulmonary nodule measuring 3 cm in diameter that doubled in volume over a 6-month period, the risk of malignancy is too high. Frequently, in the clinical practice, the cases present with cancer probability ranging between those two extremes.

Currently, the recommendation^(18)^ for estimating the pretest malignancy probability in all patients with solitary pulmonary nodule still remains valid, either qualitatively, on the basis of clinical evaluation, or quantitatively, by means of validated models.

The Bayesian analysis may be used to estimate the cancer probability (pCa) in cases of indeterminate solitary pulmonary nodules^(19)^. The principle consists in utilizing likelihood ratios (LR) in a range of clinical and radiological variables associated with the solitary pulmonary nodule. The calculation of the LR for a determined feature is the following^(3)^:

$$\mathrm{LR}=\frac{\mathrm{number}\mathrm{of}\mathrm{malignant}\mathrm{nudules}\mathrm{with}\mathrm{characteristic}}{\mathrm{number}\mathrm{of}\mathrm{benign}\mathrm{nodules}\mathrm{with}\mathrm{the}\mathrm{characteristic}}$$

A LR = 1.0 represents 50% of chance of malignancy, LRs < 1.0 indicate a benign lesion, while LRs > 1.0 indicate a malignant process. Table 2 demonstrates the LR for some clinical and radiological characteristics.

**Table 2:** Likelihood ratios (LR) for clinical and radiological characteristics of solitary pulmonary nodules

Characteristic		LR
Wall thickness (mm)	> 16	37.97
	> 4–16	0.72
	≤ 4	0.07
		
Size (cm)	> 3.0	5.23
	2.1–3.0	3.67
	1.1–2.0	0.74
	≤ 1.0	0.52
		
PET (SUVmax)	> 2.5	4.30
	≤ 2.5	0.04
		
Age (years)	> 70	4.16
	50–70	1.90
	30–39	0.24
	20–29	0.05
		
Growth pattern (days)	> 465	0.01
	7–465	3.40
	< 7	0
		
CT delayed enhancement (UH)	> 15	2.32
	≤ 15	0.04
Irregular margin		5.54
History of cancer		4.95
Active smoking		2.27
Non smoking		0.19
Indeterminate calcification at CT		2.20
Location in upper or middle lobe		1.22
Smooth margin at CT		0.30
Benign calcification pattern at CT		0.01

Adapted from Winer-Muram^((8)^

With the LR values, the chance of malignancy (Odds_ca_) is calculated:

$${\mathrm{Odds}}_{\mathrm{ca}}=\text{LR prevalence}\times \text{LR size}\times \text{LR margin}\times \text{LR smoking}\times \text{LR calcification}$$

LR prevalence corresponds to the local prevalence of malignant nodules. From the obtained malignancy chance, the pCa is calculated.

$$\mathrm{pCa}=\frac{{\mathrm{Odds}}_{\mathrm{ca}}}{\left(1+{\mathrm{Odds}}_{\mathrm{ca}}\right)}$$

Another validated model^(20)^ was developed on the basis of a study with 629 patients with indeterminate pulmonary nodules measuring between 0.4 and 3 cm in diameter detected at chest radiography. By utilizing logistic regression analysis in a series of clinical and radiological variables, the authors have identified six independent predictors of malignancy (age, smoking, history of cancer > 5 years before the nodule detection, diameter, spiculated margins and location in the upper lobe). The probability of cancer is obtained in accordance with the equation including the three clinical variables and three radiological variables:

$$\mathrm{Probability}\mathrm{of}\mathrm{malignancy}=\frac{e^{x}}{\left(1+e^{x}\right)}$$

where: *x* = -6.8272 + (0.0391 x age) + (0.7917 x *smoking*) + (1.3388 x *cancer*) + (0.1274 x *diameter*) + (1.0407 x spiculated margin) + (0.7838 x *location),* where: *e* corresponds to the basis of the natural logarithm, *age* is the patient's age in years, *smoking* = 1 if smoking or formerly smoking patient (if contrary = 0), *cancer* =1 if a history of cancer was present > 5 years before the nodule detection (if contrary = 0) and *location* in the upper lobe = 1 (if contrary = 0).

The selection of the management of the patient with solitary pulmonary nodule is complex and depends on a range of factors such as, for example, the clinical and radiological probability of cancer, risks of the procedures (biopsy/surgery), clinical conditions, local experience and the individual's preference^(18)^.

Classical studies^(21-23)^ of decision analysis models have suggested that the best strategy depends directly of the initial probability of a benign or malignant origin of the nodule. In patients with low probability of malignancy (< 3%), the greatest benefit was demonstrated with watchful waiting, i.e., serial radiographic examinations to determine whether the nodule remained stable, or the nodule volume had doubled within 2 years. On the other hand, in cases with high probability (> 68%), surgery became the preferred method for defining the cause and, at the same time, being the standard treatment at less advanced stages of lung cancer. In cases with intermediate probability, biopsy was the method of choice, but with the disadvantage of exposing the patient with a benign nodule to the potential risks of an invasive method and, many times, culminating in non-diagnostic or potentially false-negative results. It is important to highlight that those old studies did not include more advanced imaging modalities for characterization of pulmonary nodules in their analyses.

## CONSIDERATIONS ABOUT ^18^F-FDG PET/CT

PET is a nuclear medicine imaging method that allows for a noninvasive evaluation of a range of biological processes^(24)^. The hybrid apparatuses (PET/CT), idealized in the middle of the 1990s, became commercially available at the beginning of 2001, and from then on the development of this modality compares to the development of magnetic resonance imaging in the decades of 80s and 90s^(25)^.

The PET principle is similar to that of conventional scintigraphy, but with some particularities that make it a unique imaging method. The radioactive tracers utilized in this modality are positron emitters, i.e., an elementary particle with the same mass and charge magnitude of an electron, but with a positive charge. They are formed from nuclides with excess of protons in relation to the number of neutrons, therefore away from the stability range. The proton emitted from an unstable nucleus goes through some millimeters up to interact with an electron, in a process named annihilation. In this phenomenon, the electron and proton mass is converted into two γ rays traveling in opposite directions (approximately 180°) with an energy of 511 keV. The PET systems record an event at the moment when, within a determined time window, the two γ rays reach opposite detectors, forming a projection line. The informations generated by several pair of detectors are reconstituted and generate the tomographic images^(26,27)^.

Currently, many positron emitter radionuclides are available. While some of them are produced by nuclear generators (^68^Ga and ^82^Rb), others are obtained by means of cyclotrons (^11^C, ^13^N, ^15^O and ^18^F)^(26)^. The most widely utilized radiopharmaceutical is ^18^F-FDG, a glucose analog that bind to ^18^F, with a physical half-life of approximately 110 minutes^(26)^. Such a radiotracer enters the cells through membrane receptors (GLUT) and, once in the cytoplasm, it is converted into ^18^F-FDG-6-phosphate, becoming trapped in metabolically active cells since it does not follow the subsequent intracellular glucose metabolism route^(28)^. The ^18^F-FDG uptake by a cell is proportional to its metabolic activity, hence its wide applicability in a range of neoplasms^(28-30)^.

In the last decade, the development of hybrid PET/CT apparatuses has allowed for joining in a single procedure the fusion of high anatomical resolution information with its corresponding biological behavior. The addition of the molecular information provided by PET to CT is quite advantageous, since metabolic alteration occurs earlier than the morphological one^(31)^. Additionally, the CT incorporation into PET allows for procedures with shorter images acquisition time, as well as serves as a parameter for correcting the attenuation at the emission images^(26-28)^.

Quality PET/CT scans should meet a series of prerequisites such as obtaining relevant clinical information, appropriate preparation of the patient, periodical equipment quality control, images interpretation and reporting^(32)^. A recent study demonstrated a lack of standardization of the administered ^18^F-FDG activities in different Brazilian institutions, justifying the necessity of officially establish a reference value to be adopted^(33)^.

Generally, the images interpretation is qualitative, and a focal increase in ^18^F-FDG uptake above the blood pool is considered to be abnormal. However, in order to minimize interpretation errors, the knowledge about the patterns of radiopharmaceutical physiological distribution is fundamental, as is the knowledge about physiological variants and potential benign diseases^(34)^. A quantitative method frequently utilized is the standardized uptake value (SUVmax), whose calculation corresponds to^(32)^:

$$\mathrm{SUVmax}=\frac{\frac{{\mathrm{Act}}_{\mathrm{voi}}\left(\frac{\mathrm{kBq}}{\mathrm{mL}}\right)}{{\mathrm{Act}}_{\mathrm{administered}}\left(\mathrm{MBq}\right)}}{\mathrm{Weight}\left(\mathrm{kg}\right)}$$

where: *Act_voi_* corresponds to activity measured in the volume of interest; *Act_administered_* is the administered activity corrected by the decay at the beginning of the images acquisition.

Many studies have demonstrated the diagnostic performance of ^18^F-FDG PET and PET/CT in the characterization of solitary pulmonary nodules in different populations.

## CONCLUSION

The introduction of imaging methods such as chest radiography and CT resulted in great advances in the management of patients with pulmonary conditions. Pulmonary nodules whose morphological characteristics many times overlap between benign and malignant processes, represent a diagnostic challenge and have been increasingly identified. The knowledge about epidemiology, morphological characteristics and methods to estimate the likelihood of malignancy became fundamental in the investigation of patients with solitary pulmonary nodule.

The difficult characterization of many of the solitary pulmonary nodules has determined a special field attracting attention to other techniques such as, for example, ^18^F-FDG PET/CT, dynamic contrast-enhanced CT and magnetic resonance imaging. The best noninvasive way to stratify risks in this scenario is still a subject of discussion aimed at making decisions with a better cost-benefit ratio for both the patients and the health system.
